# Testing 3D printed biological platform for advancing simulated microgravity and space mechanobiology research

**DOI:** 10.1038/s41526-022-00207-6

**Published:** 2022-06-03

**Authors:** Giulia Silvani, Peta Bradbury, Carin Basirun, Christine Mehner, Detina Zalli, Kate Poole, Joshua Chou

**Affiliations:** 1 School of Biomedical Engineering, Faculty of Engineering and Information Technology, University of Technology Sydney, Ultimo, NSW Australia; 2 Institut Curie, Paris Sciences et Lettres Research University, Mechanics and Genetics of Embryonic and Tumoral Development Group, Paris, France; 3 Department of Physiology and Biomedical Engineering, Mayo Clinic, Jacksonville, FL USA; 4 Institute of Continuing Education, University of Cambridge, Camridge, UK; 5 EMBL Australia Node in Single Molecule Science, School of Medical Sciences, Faculty of Medicine & Health, Sydney, NSW Australia

**Keywords:** Biomedical engineering, Protein-protein interaction networks, Molecular medicine, Targeted therapies

## Abstract

The advancement of microgravity simulators is helping many researchers better understanding the impact of the mechanically unloaded space environment on cellular function and disfunction. However, performing microgravity experiments on Earth, using simulators such as the Random Positioning Machine, introduces some unique practical challenges, including air bubble formation and leakage of growth medium from tissue culture flask and plates, all of which limit research progress. Here, we developed an easy-to-use hybrid biological platform designed with the precision of 3D printing technologies combined with PDMS microfluidic fabrication processes to facilitate reliable and reproducible microgravity cellular experiments. The system has been characterized for applications in the contest of brain cancer research by exposing glioblastoma and endothelial cells to 24 h of simulated microgravity condition to investigate the triggered mechanosensing pathways involved in cellular adaptation to the new environment. The platform demonstrated compatibility with different biological assays, i.e., proliferation, viability, morphology, protein expression and imaging of molecular structures, showing advantages over the conventional usage of culture flask. Our results indicated that both cell types are susceptible when the gravitational vector is disrupted, confirming the impact that microgravity has on both cancer and healthy cells functionality. In particular, we observed deactivation of Yap-1 molecule in glioblastoma cells and the remodeling of VE-Cadherin junctional protein in endothelial cells. The study provides support for the application of the proposed biological platform for advancing space mechanobiology research, also highlighting perspectives and strategies for developing next generation of brain cancer molecular therapies, including targeted drug delivery strategies.

## Introduction

The presence of extracellular mechanical forces are central to developmental biology, tissue homeostasis and disease onset in humans^1^. The process of mechanotransduction begins at the cellular level and is mediated by molecular mechanotrasducers complexes that upon sensation of mechanical cues induce activation and inhibition of biochemical signaling pathways that ultimately determine cell mechanical properties and functionality^2^. Whilst it has long been demonstrated and widely accepted that mechanical forces play a critical role in regulating human (patho)physiology, the reverse should also hold true, i.e., the absence or reduction of extracellular forces (known as mechanical unloading) also affects cell function and overall tissue homeostasis. Clear examples of the effects that mechanical unloading plays on human functions are the observed, apparent changes that occur when astronauts encounter the zero-gravity environment of space, including reduced bone and muscle density, increased risk of cardiovascular disease, vision problems and a compromised immune system^3–7^. As such, many scientists are putting great effort in understanding the underlying molecular mechanisms involved in mechanically unloaded diseases. However, conducting in vitro space research missions on the International Space Station is still in its infancy and is also often not a financially viable option. Hence, researchers have developed microgravity (µG) simulators capable of producing near zero-gravity environments, such as the random positioning machines (RPM) and clinostats^8,9^. In recent years, the advancement of µG simulators has helped and accelerated the discovery of several molecular candidates, which have been proposed as mechanosensing molecules involved in cell adaptation and survival to the μG environment, including adhesion receptors, extracellular ligands, ion channels, growth factor receptors, and membrane proteins^10^. Understanding how these molecules modulate, transduce, and integrate a “lack of gravitational force” represents a possible approach to reveal mechanotransduction processes driving not only space-related pathology but also severe terrestrial diseases that would go otherwise undetected in normal gravity condition. For instance, cancer cells have been found to be highly sensitive to the mechanical unloaded environment, responding by modulating malignancy, proliferation, chemoresistance, and cell survival^11–14^. Therefore, further investigation in the emerging field of space mechanobiology will bring meaningful information to be considered for next generation medical treatment and intervention on Earth.

However, performing microgravity experiments on Earth using simulators introduces some unique practical challenges for biological experimentation that limit the progress of the research^15^. In particular, the RPM simulator allows the study of cell behavior under disrupted gravitational force by randomly rotating the X, Y, and Z axis upon which a biological sample sits, thus inducing a constant change in the gravity vector calculated to be near zero gravity (10^−3^ *g*)^9^. Conventional use of RPM simulator utilizes cell culture plastic, like flasks, that need to be filled entirely with expensive media (~50 mL for the T25 flask) to avoid the addition of shear stress that may arise from air bubbles during rotation. Additionally, the large volume now covering the cells may limit gas and oxygen diffusion through the flask, resulting in confounding parameters that also need to be tested and appropriately accounted for. A final concern among μG researchers is the use of cell culture plastic welled plates, as aseptically sealing the plates to ensure RPM rotational experiments free of leakages, air bubbles, and contamination, is challenging. Thus, to further advance μG research and facilitate experiments in a laboratory setting, new economically accessible technologies and reliable tools are needed to provide alternatives for researchers.

Microfluidics and 3D lab-on-a-chip (LOC) approaches have been developed as highly valuable miniaturized platforms that improve high-throughput analysis and allow cost-effective, reproducible, disposable chips that can be fabricated in mass production^16^. LOC systems are typically fabricated by polydimethylsiloxane (PDMS) replica molding^17^ and are suitable for applications in the biomedical field, including cell analysis, cell manipulation and drug discovery, as they demonstrated to be highly biocompatible and gas permeable^18–21^. With the advancement of 3D printing technology, LOCs systems are further expanding their versatility by offering precision and high-resolution design to establish a fast and cheaper alternative approach to conventional lithography processes^22–24^.

Here, we showcase the hybrid production of a biological platform that utilizes 3D printing technology, using sacrificial material, coupled with PDMS microfluidic fabrication process to develop reusable user’s friendly device, henceforth called microgravity-on-a-chip (MOC), that facilitates reliable and reproducible RPM-simulated µG cellular experiments. The aim of the present study was then to demonstrate the versatility and compatibility of MOC with a vast array of biological assays, i.e., proliferation, viability, morphology, protein expression and imaging of molecular structures and to characterize the system with two cell lines of different phenotype and functionality in the contest of brain cancer research. We characterized the system using A-172 Glioblastoma cells and HUVEC cells to respectively model the tumor and the endothelial monolayer, mimicking the vascular barrier observed in vivo. Indeed, in addition to the direct impact on tumor on set and progression, μG may also advance our understanding in treatment development and drug delivery strategies, as the main challenge in most cancer therapies is the ineffective delivery of chemotherapeutics across blood vessels^25–28^. Lining the blood vasculature are endothelial cells that form a continuous physical and biological barrier via intercellular adherent junctions that regulate both vascular integrity and solute permeability, including chemotherapeutics. Importantly, these junctional structures have been shown to be highly sensitive to changes in the mechanical environment with recent μG studies identifying that a mechanically unloaded environment affected endothelial cell function, viscoelastic properties, and proteome^29–32^. Thus, using the proposed MOC system, we questioned if μG can be exploited as an approach to facilitate the discovery of molecular targets that determine Glioblastoma aggressiveness, including vascular junctional architecture, to ultimately develop brain cancer molecular therapies and targeted drug delivery strategies.

## Methods

### Fabrication of microgravity-on-a-chip (MOC)

The MOC was designed using CAD software (Solidworks, Dassault Systèmes) to generate a.STL file consisting of four circular chambers (6 mm in diameter) connected in series by a microfluidic channel. To realize different MOCs, 4 CAD designs were prepared with different chamber heights, namely 0.8, 2, 2.7 and 3.5 mm. A Cellink Biox 3D bioprinter (Cellink, Sweden) with a 27 G nozzle was used to extrude Pluronic F127 40% in a standard 100 mm petri dish (Corning, Australia) at 5 mm/s under 160 kPa at 26 °C. The printing parameters include a grid infill pattern with 60% density with printed fibers of 0.1 mm each. After the designed construct was printed, PDMS at a ratio of 10:1 was casted into the petri dish carefully covering the entire printed structure. The petri dish was then placed overnight in an oven at 35 °C to let the PDMS polymerized and subsequently peeled off and cut to separate each device. The mold was then washed carefully with distilled water to remove residual Pluronic F127. Inlet and outlet holes were punched before the device was plasma bonded to a glass slide. The MOC was sterilized with UV light for 30 min and washed twice with Dulbecco Phosphate Buffered Saline (PBS, Sigma Aldrich, Australia) using 1 mL syringe and tygon tubing (John Morris Scientific, Cat. NO ND-100-80, Australia).

### Cell maintenance and cell seeding into the MOC

Human Glioblastoma cells (A-172) were purchased from Sigma Aldrich, Australia (Cat. No. 88 062 428) and cultured with high-glucose Dulbecco’s Modified Eagle Medium (DMEM) (Thermofisher, Cat. No. 11 965 084, Australia) supplemented with 10% fetal bovine serum (Thermofisher, Australia) and 5% penicillin-Streptomycin (Thermofisher, Cat. No. 15 140 122, Australia) and kept in humidified incubator at 37 °C with 5% CO_2_. Media was changed every 3–4 days and the cells were passaged upon reaching 90–95% confluence. Cells were tested for mycoplasma contamination and found negative. HUVECs were purchased from Lonza, Australia (Cat. No. CC-2517) and grown in humidified incubator at 37 °C and 5% CO_2_ using basal medium-2 (EBM-2) supplemented with endothelial growth medium (EGM-2) BulletKit from Lonza, Australia (Cat. No. CC- 3162). To ensure the expression of key endothelial proteins, HUVEC cells were only used between passage numbers 2–5. Once the cells reached 80–90% confluency, they were washed twice with PBS and detached using Trypsin-EDTA solution (Sigma Aldrich, Australia). Cell suspension was collected and centrifuged at 180 × *g* for 2 mins and the supernatant was discarded. Cells were then resuspended in the corresponding culture medium at an average concentration of 1 × 10^5^ cells/mL for both cell types. Prior to cell seeding, MOCs were washed with PBS and functionalized with either Collagen Type I (Thermofisher Scientific, Cat. No. A1048301, diluted in PBS 1:20) for Glioblastoma cells (A-172) or fibronectin (1:50 in PBS, Sigma Aldrich, Cat. NoF1141, Australia) for HUVEC. Cell suspensions were injected into the MOC using tygon tubes. Cells were then incubated overnight under static condition, and media changed prior to μG experiments. Importantly, for junction opening evaluation HUVECs were grown into the MOC platform to confluency to ensure the complete maturation of junction and barrier functionality.

### Microgravity assays

A Random Position Machine (RPM) (EXPLOR Space Technologies, Australia) was used to simulate the μG condition experience in space. The system was a desktop-size 3D clinostat placed in a incubator and worked by changing the X, Y, and Z axis of the arm, inducing random changes to the gravity vector orientation thus resulting in an average gravity zero vector over time. The MOC containing the cells was carefully placed in the center of the arm to avoid any points of the sample being affected by residual centrifugal acceleration. Relative static control (1 G) was placed in the same incubator. All μG experiments lasted maximum 24 h unless otherwise specified.

### Viability and counting cells procedures

To verify both the biocompatibility of MOC platform and assess viability of Human Glioblastoma A-172 and HUVEC cells after μG experiments, live imaging using LIVE/DEAD Viability/Cytotoxicity Kit (Sigma-Aldrich, Cat. No. L3224) was performed as per manufactures instructions. Briefly, fluorescent calcein-AM (2 μM) and red-fluorescent ethidium homodimer-1 (EthD-1) (4 μM) were gently injected into the MOC and incubated for 15 min at 37 °C and humidify atmosphere. MOCs were then washed with PBS and imaged using EVOS M5000 microscope (Invitrogen). To evaluate proliferation, cells were detached from the MOC using Trypsin-EDTA, collected, centrifugated and counted using a Hemocytometer. Trypan Blue 0.4% (Thermofisher Scientific, Cat. No. 15250061) was used to determine the percentage of viable cells present in the suspension.

### Fluorescence staining and imaging

Visualization of actin cytoskeleton filaments and endothelial adherent junctions was assessed by immunofluorescence imaging with EVOS M5000 microscope. Media from the MOC was aspirated and cells were washed with PBS prior to being fixed with 4% paraformaldehyde (PFA) for 20 min at RT. Cells were washed to remove residual PFA and permeabilized using 0.1% Triton X-100 for 10 min. Cells were then blocked for 1 h with 1% Goat serum (Sigma Aldrich, Cat. No. G9023) and immunostained for VE-Cadherin (1:1000; 1% Goat serum; Abcam, Cat. No. ab33168) and incubated overnight at 4 °C. Cells were then co-incubated with Goat anti-rabbit Alexa fluor 488, (1:200; PBS; Abcam, Cat. No. ab150077) and TRITC-Phalloidin (1:10000; Sigma- Aldrich, Cat. No. P1951) for 1 h in the dark. Nuclei were stained with DAPI.

### Cell morphological analysis

Morphological analysis was performed on Human Glioblastoma A-172 and HUVEC cells to evaluate parameters such as area (A), shape index (SI), and Tortuosity index (TI) as described previously^33^. The cell outline was manually extracted using ImageJ software by selecting the peripheral actin filaments in fluorescence images. A binary image was created and used for automatic quantification of morphological parameters, such as A, SI and perimeter. Also, the major and minor axes of the equivalent ellipse for the cell outline were determined. The SI value is a measurement of cell roundness, from a perfect spherical shape (SI = 1) to an elongated shape (SI = 0) and is defined as follows:

$${{{\mathrm{SI}}}} = 4\pi {{{\mathrm{A}}}}/{{{\mathrm{P}}}}^2,$$

where A is the cell area and P the cell perimeter. Another parameter, known as tortuosity index (TI), assesses and quantifies whether cell approaches a smooth circular or elliptical profile (TI = 1) or an irregular star shape profile (TI > 1). The TI is defined as follows:

$${{{\mathrm{TI}}}} = {{{\mathrm{P}}}}/{{{\mathrm{P}}}}^\prime ,$$

where P is the cell perimeter and P′ the equivalent ellipse perimeter of the cell. Morphological parameters were measured from 50 cells in 8 different fluorescence images for each experimental condition.

### Quantitative in-cell western assay using MOC

Quantitative analysis of proteins expression was carried out using the rapid and high-throughput In-Cell Western™ (Odyssey®) as per manufactures instructions. Briefly, cells were seeded in MOCs at a concentration of 1 × 10^5^ cells/mL and cultured overnight. The cells were then subjected to μG for 24 h and immediately fixed as described above. Cells were permeabilized with 5 washes of 0.1% Triton X-100 (v/v)/PBS for 5 min per wash. Cells were then blocked in Odyssey Blocking Buffer for 90 min at RT and incubated overnight at 4 °C with primary antibodies against VE-Cadherin (1:1000) or active (non-phosphorylated) Yap-1 (1:200; Abcam, Cat. No. ab205270) and Vinculin (1:400; Sigma-Aldrich, Cat. No. V9131). Cells were washed with 0.1% Tween-20 (v/v)/PBS for 5 min and stained with IRDye secondary antibodies (1:200), 1 hr at RT. Finally, the MOCs were scanned with the Odyssey CLX system (Li-Cor Biosciences) equipped with a near-infrared light technology for signal detection, adapting the scanning process for sample on glass slide with at a distance of 1 mm. Signal intensity was quantified with Image Studio software (Version 4.0; Li-Cor) according to the manufacturer’s instructions.

### Image analysis

#### Junction protein and gap formation evaluation

To evaluate the effect of μG at the molecular level in HUVECs, VE-Cadherin pattern was followed and analyze using MATLAB software (MathWorks) at 2 different conditions: sub-confluent and confluent condition. For junction protein development evaluation, a sub-confluent monolayer of HUVECs was subjected to 24 h of μG and then imaged following fluorescence staining procedures. The raw intensity profile of the junction along the entire cell perimeter and the average intensity of multiple random regions within cell membrane were determined for 50 cells. The intensity profile of junction protein was calculated as the VE-Cadherin intensity subtracted by the average intensity of cytoplasm and then normalized pixel by pixel by the intensity along the cell border. The corrected intensity profile was then plotted with the positive intensity indicating junctional VE-Cadherin formation and the negative intensity representing gap between cells. The reported staining percentage of VE-Cadherin was calculated as the percentage of pixels with positive intensity values along the entire cell border. For junction protein opening quantification, a matured confluent monolayer of HUVECs was subjected to μG condition for 24 and 48 h and imaged following fluorescence staining procedures for VE-Cadherin protein at each time point. From the cell membrane staining, the space between cells were circled, counted, and measured by area using ImageJ for 30 different fluorescence images for each experimental condition. Results were presented as normalized percentage of the opened area and as distribution of gaps in number and dimension.

### Actin stress fibers remodeling

Changes in actin stress fibers organization were evaluated by performing line scans using ImageJ and analyzing the resulting fluorescence profile, as already described^34^. Briefly, lines were drawn within 50 individual cells chosen randomly within the MOC chamber, along the smaller axis, perpendicular to stress fibers. After image correction for background, the resulting fluorescence intensity profiles were filtered and analyzed for the number of peaks above an arbitrary baseline and at a defined distance from neighbors. In this way, two neighboring top-values were considered as two separate peaks only when the distance between them was equal or higher than 3 μm. For each cell, the number of peaks was divided by the length of the scan line, resulting in the density of actin stress fibres. A chart box is then plotted, showing the median density, scatter data points and error bars for each experimental conditions and cell lines.

### Statistical analysis

The statistical analysis of the data was performed using one-way analysis of variance (ANOVA) with Turkey’s test for multiple comparisons, using GraphPad Prism (v 7.04). Average values of at least three independent experiments ± SEM are showed for each of the assays. Morphological parameters and junction evaluation were measured from 50 cells in 8 different fluorescence images for each experimental condition while gaps quantification has been performed on 30 different fluorescence images for each experimental condition. *P* value is reported for statistical significance. Comparisons between samples were considered to be statistically significant if the *p* value was *p < 0.05, **p < 0.01, ***p < 0.001, **** *p* < 0.0001.

### Reporting summary

Further information on research design is available in the Nature Research Reporting Summary linked to this article.

## Results

### Development and optimization of MOC platform

**Fig. 1:**
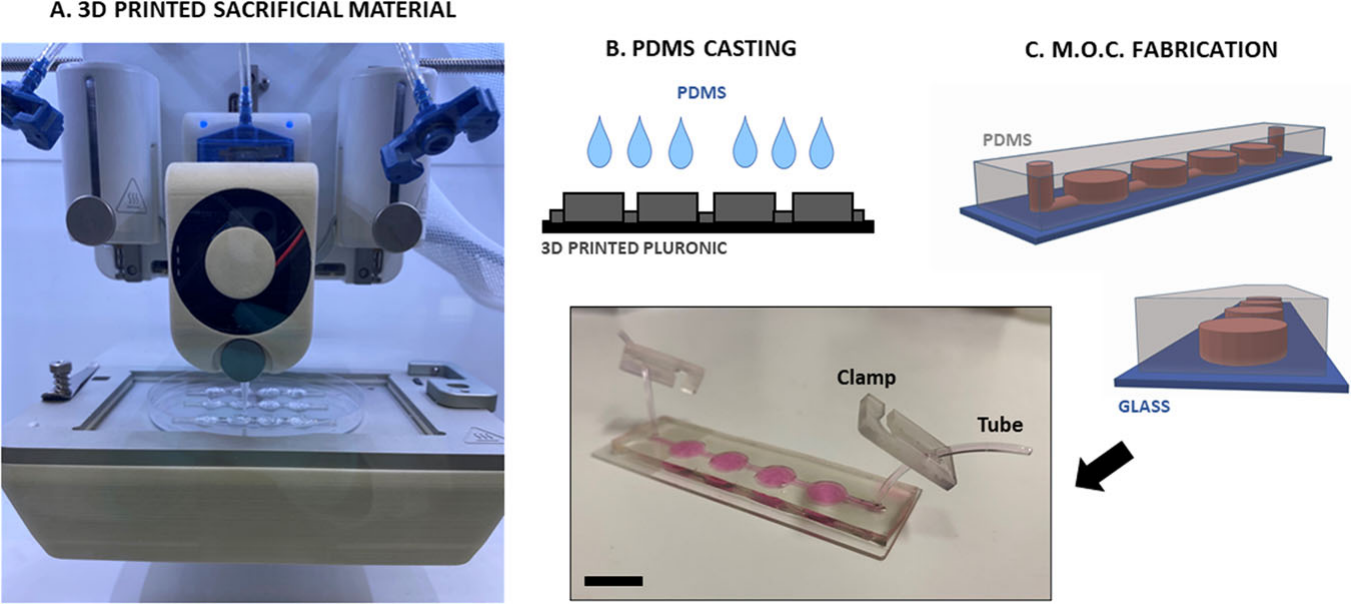
Fabrication steps for MOC. **A** Pluronic F127 40% ink is printed on a standard petri dish. **B** PDMS is casted over the printed construct and let polymerized for an overnight. **C** The mold is peeled off and bonded on a glass slide. Picture (Copyright holder: Giulia Silvani) showing the real MOC containing media with tubing and clamp. Scale bars, 1 cm.

**Fig. 2:**
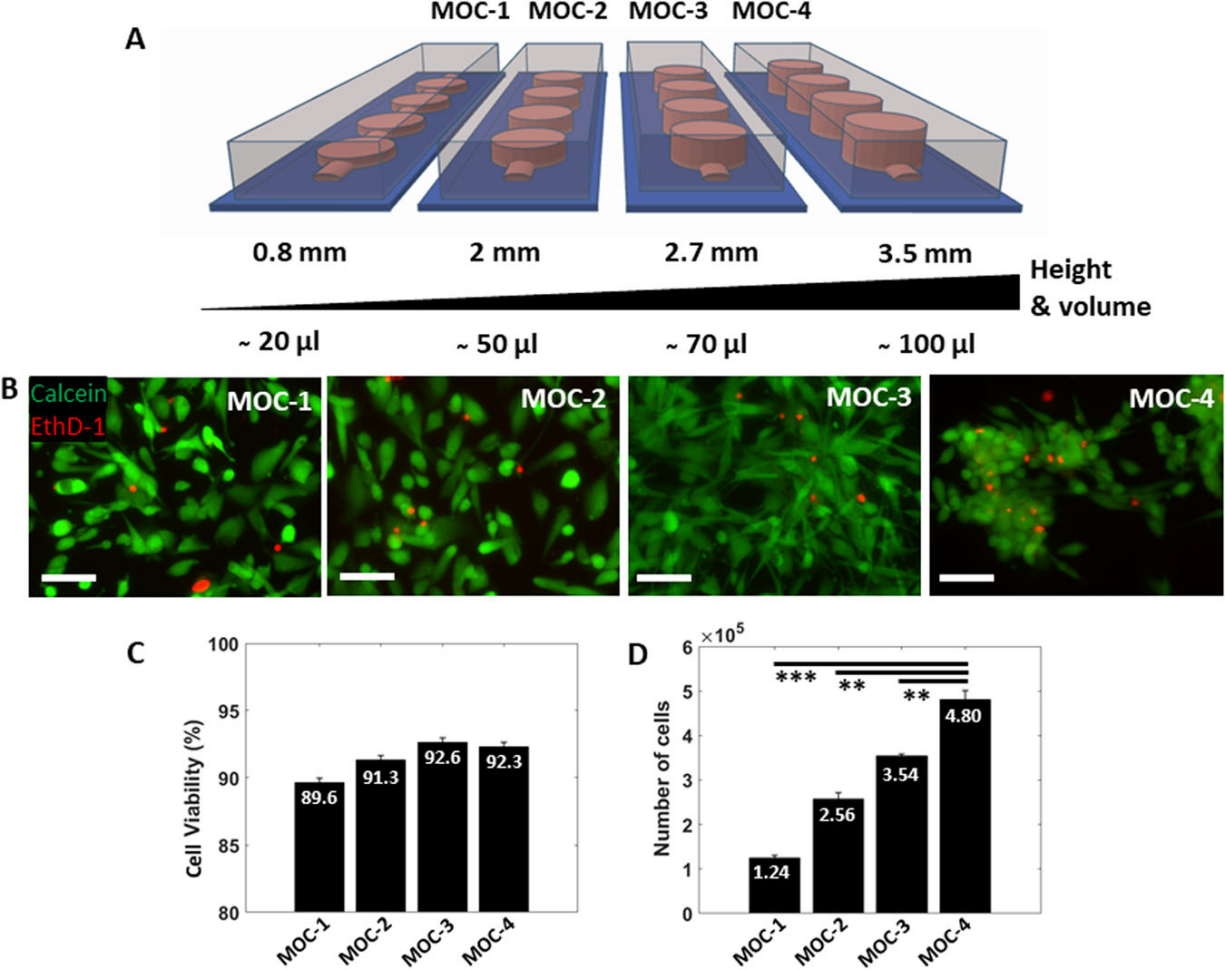
MOC biocompatibility. **A** Sketch showing the 4 different MOCs in height and volume, namely 0.8, 2, 2.7, 3.5 mm corresponding to ~20, 50, 70 and 100 μL. **B** Live cell imaging of A-172 glioblastoma cells seeded within the respective MOC devices for 48 h. Cells were stained with the Live/Dead stain where cells shown in green (Calcein AM) indicate live cells and those shown in red (EthD-1) indicate dead cells. **C** Graph indicates the percent of cells viable per MOC. **D** Graph shows the absolute number of cells present in each MOC following 48 h of growth. Scale bars, 50 µm. Data are shown as the mean ± SEM (*n* = 3). Asterisks are reported for cases with statistical significance: ** MOC-4 *Vs* MOC-3: *p* = 0.004, MOC-4 *Vs* MOC-2: *p* = 0.009, *** MOC-4 *Vs* MOC-1: *p* = 0.001. The statistical analysis of the data was performed using one-way analysis of variance (ANOVA) with Turkey’s test for multiple comparisons.

This study combined the precision and manufacturing of 3D printing technology coupled with conventional PDMS microfluidic fabrication to establish a lab-on-a-chip system and provide researchers with a tool for advancing simulated μG experiments, henceforth called microgravity-on-a-chip (MOC). Using Pluronic F127 40% as a sacrificial material, the MOC design was printed and fabricated as demonstrated in Fig. 1. The MOC consisted of 4 circular chambers connected in series by a microfluidic channel creating a closed and sealed environment that could be filled with media and cells via syringe and tubing. The fabrication process allowed for the chamber to be optimized in order to achieve the best culture condition as a combination of nutrient intake and reasonable number of collected cells for biological assays. Specifically, 4 different heights in CAD design have been chosen to obtain MOCs with same surface area but different volumes, namely 20, 50, 70, and 100 μL, MOC-1, MOC-2, MOC-3, MOC-4 respectively (Fig. 2A). A-172 glioblastoma (GBM) cells were seeded within each MOC platform at the same concentration (1 × 10^5^ cells/mL). Live&Dead imaging together with Trypan blue viability assays, were performed after 48 h to determine cell viability and proliferation. All MOCs expressed high cell viability, indicated by the green fluorescence signal within the cellular cytoplasm representing intracellular esterase activity (Fig. 2B) and confirmed by the Trypan Blue viability assay, showing 90–93% viability range (Fig. 2C). The volume of the chamber determined the number of cells initially seeded, which increased with the increased volume. As expected, when cells were collected after 48 h and counted, MOC-1, designed with equal surface area but less media volume, exhibited the lowest number of cells while the MOC-4 device showed a significant increase in cell number when compared to MOC-1, MOC-2, and MOC-3 (Fig. 2D). Notably, cells seeded in MOC-4 were shown to aggregate and form spheroids within the chambers after 48 h (Fig. 2B), representing a possible drawback when analyzing cell morphology and protein expression at different time points. Based on these results, we selected MOC-3 platform for subsequent evaluations in μG experiments as it showed a reasonable total number of cells collected, i.e., 3.5 × 10^5^ cells, that is required for biological assays at different time points.

### Exposure to microgravity reduces proliferation and alters cellular morphology

**Fig. 3:**
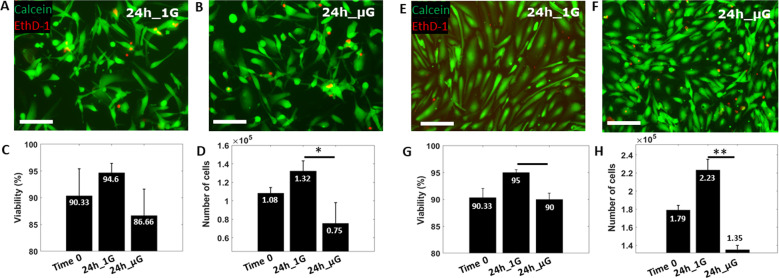
Viability and proliferation assay. Live cell images of Glioblastoma A-172 and HUVEC cells, showing Calcein AM in green and ethidium homodimer-1 (EthD-1) in red after 24 h of gravity (1 G) (**A, C**) and μG (**B, D**) conditions respectively. Cell viability (**E, G**) and proliferation evaluation (**F, H**) for A-172 and HUVEC cells respectively, at different experimental condition. Images have been acquired with ×40 magnification lens thus they refer to a small section of the entire chamber. Scale bars, 100 µm. Data are shown as the mean ± SEM (*n* = 3). Asterisks are reported for cases with statistical significance: * GBM number of cells: 24 h 1 G *vs* 24 h μG, *p* = 0.03, * HUVEC viability: 24 h 1 G *vs* 24 h μG, *p* = 0.017 ** HUVEC number of cells: 24 h 1 G *vs* 24 h μG, *p* = 0.0025. The statistical analysis of the data was performed using one-way analysis of variance (ANOVA) with Turkey’s test for multiple comparisons.

To demonstrate the applicability of the proposed biological platform for simulated μG experiments on both cancer and healthy cells, A-172 GBM cells and HUVEC cells were seeded into the MOC platform and incubated overnight prior to experimentation. Cells were analyzed for cell viability, proliferation, and morphology at time 0 and 24 h post exposure to either 1 G control or µG conditions. Live&Dead images of both cell lines following 24 h of exposure to gravity (1 G) (Fig. 3A, E) or μG (Fig. 3B, F), showed an intense, green fluorescence signal signifying high cytoplasmic esterase activity thus suggesting high viability within the MOC chamber. This was further confirmed using the Trypan Blue viability assay showed 86–94% viability for GBM cells across all conditions with no significant difference observed (Fig. 3C). However, those GBM cells exposed to μG showed a significant reduction in total cell number when compared with 1 G control (Fig. 3D), suggesting that while GBM cell viability was unaffected by µG exposure, proliferation had been significantly reduced in a µG environment. Similarly, HUVECs exhibited a cell viability range of between 90–95% for all test conditions (Fig. 3G) and a limited proliferation under μG condition (Fig. 3H).

**Fig. 4:**
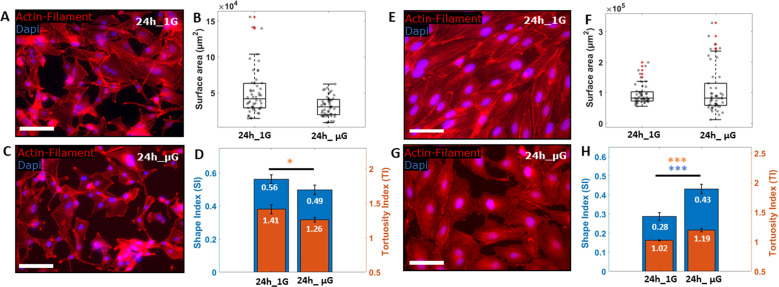
Morphology evaluation. Fluorescence images of GBM and HUVEC cells, showing actin filaments (red), VE-Cadherin (green) and DAPI (blue) after 24 h of gravity (1 G) (**A**, **E**) and μG (**C**, **G**) conditions respectively. Box plots showing the changes of surface area (**B**, **F**) and histograms presenting Shape Index (SI) together with Tortuosity Index (TI) (**D**, **H**) for A-172 and HUVEC cells respectively, at different experimental condition. Scale bars, 100 µm. Data are shown as the mean ± SEM (*n* = 50). Asterisks are reported for cases with statistical significance: * GBM SI: 24 h 1 G *Vs* 24 h μG, *p* = 0.04, *** HUVEC SI an TI: 24 h 1 G *Vs 2*4 h μG, *p* = 0.001. The statistical analysis of the data was performed using one-way analysis of variance (ANOVA) with Turkey’s test for multiple comparisons.

To further characterize the GBM and HUVEC cells response to simulated µG, we next analyzed changes to cell morphology. Cells were stained for actin filament following 24 h of exposure to either 1 G control conditions (Fig. 4A, E) or μG conditions (Fig. 4C, G). Three morphology parameters were quantified: cell area, shape index (SI) and tortuosity index (TI)^33^. Briefly, SI measures cell roundness, where an SI = 1 indicates a perfect circle and an SI = 0 represents an elongated cell; TI assesses whether cell border has a smooth profile (TI = 1) or an irregular star shape profile (TI > 1). All cells exposed to the mechanical unloaded environment showed no significant change in cell area when compared to 1 G sample (Fig. 4B, F). Interestingly, GBM cells exposed to µG showed no significant change in SI, but a significant decrease in TI was observed when compared to cells growth under 1 G environment (Fig. 4D), suggesting that while there was no change to the overall size or shape of GBM cells, they had become smoother at the cell edges. Conversely, HUVECs exposed to 24 h of µG were shown to significantly increase both SI and TI (Fig. 4H), displaying a much rounder and tortuous morphology (Fig. 4G) when compared to 1 G cells (Fig. 4E). Overall, these results indicated that both GBM and HUVEC cells were able to sense a change in the gravitational vector within 24 h and responded by suppressing proliferation activity and inducing morphological changes that did not compromise cell viability.

### Microgravity modulate mechanosensitive proteins expression

**Fig. 5:**
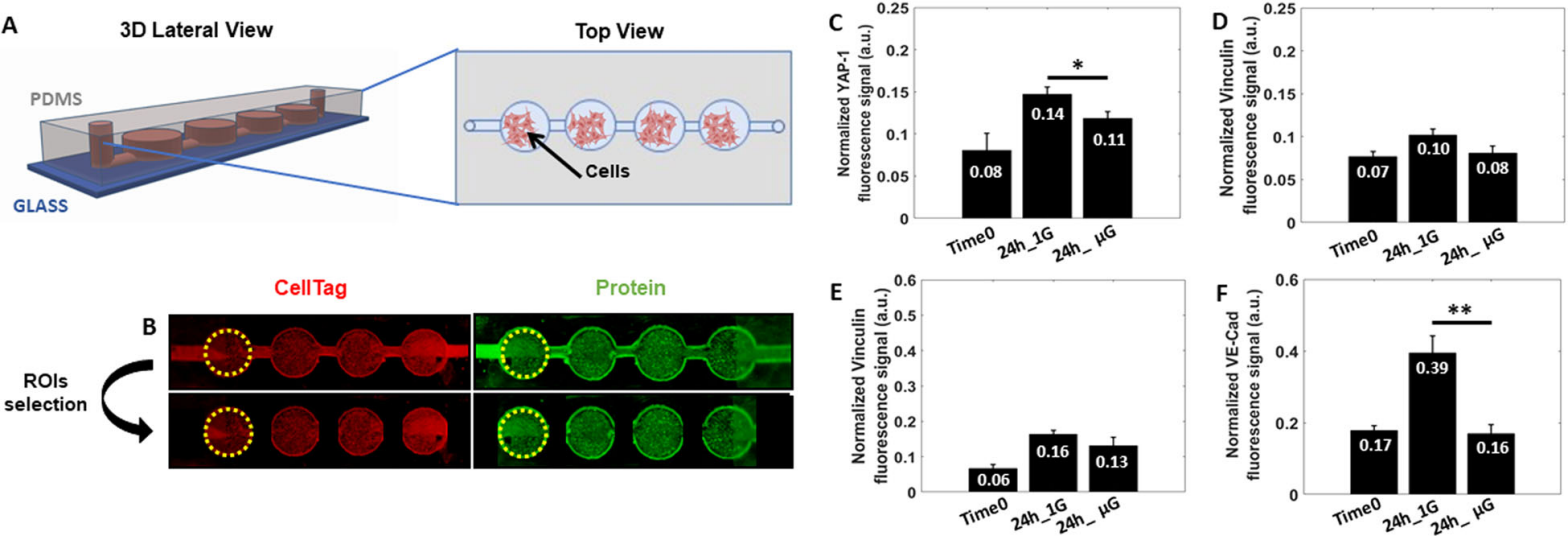
Protein expression analysis. **A** Graphical rendering of the MOC showing the 3D lateral and Top view. **B** Example of IWB plate images showing Cell Tag 700 stain, in red, for cell number normalization and IRDye secondary antibodies, in green, for protein fluorescence signal detection. A dashed yellow circle highlights the Region of Interest (ROI) selected for analysis. IWB assay for changes in Active Yap-1 (**C**) and Vinculin (**D**) proteins expression in response to 24 h of μG condition, in Glioblastoma A-172. IWB assay for changes in Vinculin (**E**) and VE-Cadherin (**F**) proteins expression in response to 24 h of μG condition, in HUVECs. Data are shown as the mean ± SEM (*n* = 3). Asterisks are reported for cases with statistical significance: * YAP-1 24 h 1 G *Vs* 24 h μG: *p* = 0.329, ** VE-Cadherin 24 h 1 G *Vs* 24 h μG: *p* = 0.013. The statistical analysis of the data was performed using one-way analysis of variance (ANOVA) with Turkey’s test for multiple comparisons.

The Odyssey In-Cell Western Blot (IWB) methodology allows quantitative measurement of protein expression using fluorescently labeled secondary antibodies along with a fluorescence scanner. In this study, the MOC was developed to be compatible with the Odyssey IWB machine as the diameter of the MOC’s circular design was the same dimension of the conventional 96 well-plate and could be directly analyzed with the default settings on the Odyssey machine by selecting the Region of Interest (ROI) as shown in Fig. 5A, B.

As a first step toward understanding mechanotransduction processes triggered by μG in both GBM and HUVECs cells, key mechanosensitive proteins have been investigated and analyzed. For instance, we sought to evaluate the impact of μG on active yes-associated protein 1 (YAP-1) and Vinculin in A-172 GBM cells, which are both known to cooperate in adhesive mechanotransduction processes regulating cell migration and proliferation^35,36^. Importantly, A-172 GBM cells subjected to 24 h of μG showed a significant reduction in active YAP-1 (22%) and a slight decrease in Vinculin (20%) protein expression compared to 1 G control (Fig. 5C, D). Same trend was followed by Vinculin expression in HUVECs after 24 h of μG exposure (Fig. 5E).

In HUVECs, the vascular endothelial cadherin (VE-Cad) junction protein is a mechanotrasducers that acts in response to external physiological forces^37^. VE-Cad localizes at cell-cell border where it complexes with other proteins to form a dynamic biological barrier that ultimately regulates vascular integrity and permeability^28^. Therefore, we next looked at VE-Cad expression in HUVECs exposed to 24 h of µG and found that those cells subjected to μG exhibited a significant reduction in VE-Cad protein expression (59%) compared to 1 G sample (Fig. 5F). Moreover, VE-Cad protein expression levels of HUVECs subjected to µG were equal to VE-Cad expression at Time 0, suggesting that exposure to µG had altered VE-Cad turn over at cell-cell junctions or delayed VE-Cad-positive junction formation and maturation^38^.

### Exposure to microgravity obstructs cell-cell junction maturation

**Fig. 6:**
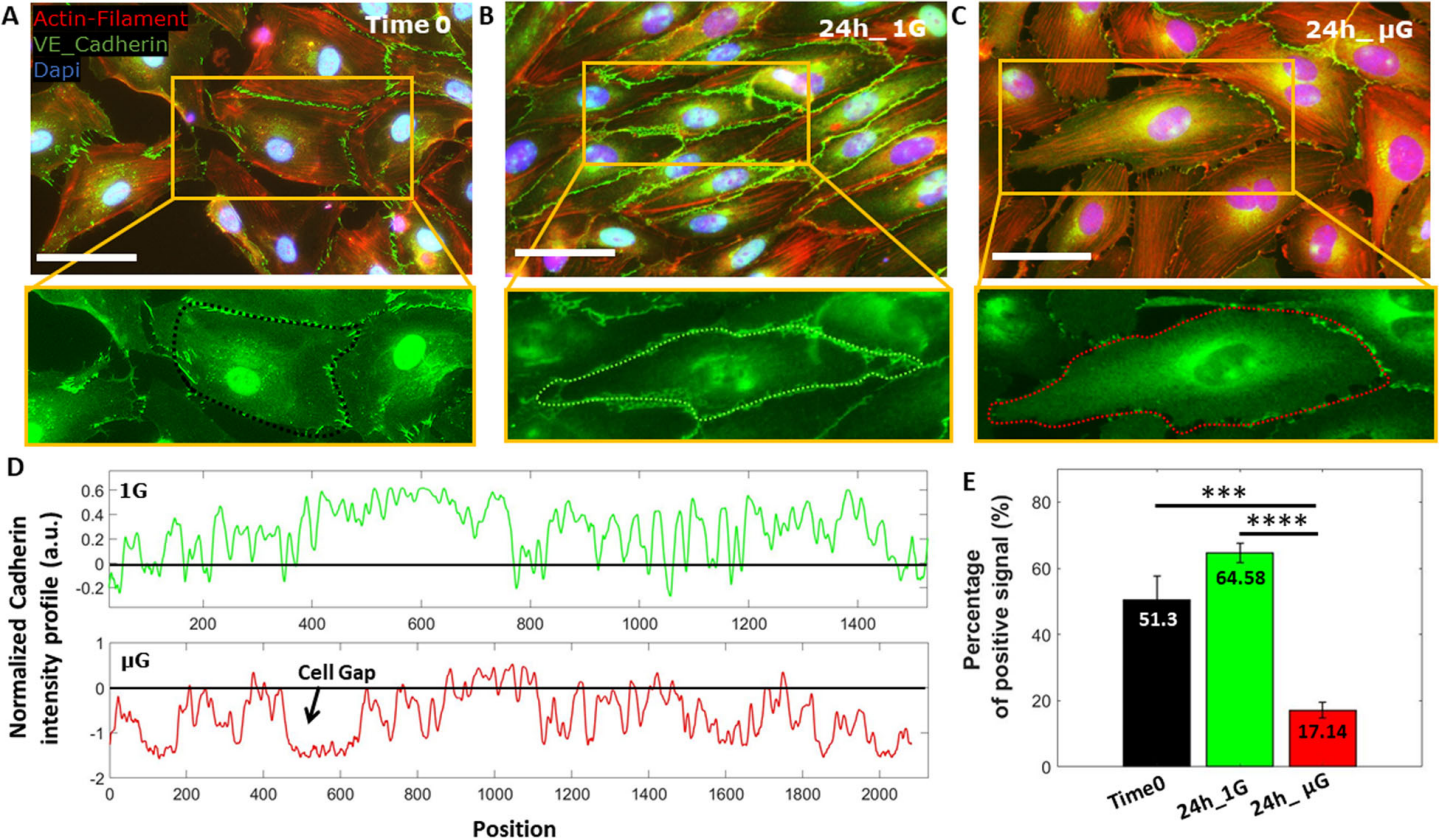
Junction development evaluation. Fluorescence images of a sub-confluent monolayer of HUVECs showing Actin-Filament. (red), VE-Cadherin (green) and Dapi (blue) at Time 0 (**A**) and after 24 h of gravity (1 G) (**B**) and μG (**C**) conditions. **D** Fluorescence. profile of VE-Cad patter along the perimeter of the cell for 1 G and μG sample. The positive intensity represents junctional protein formation while the negative intensity indicates gaps between cells. **E** Histogram showing the percentage of positively stained VE-Cad along the entire cell border for all experimental conditions. Scale bars, 50 µm. Data are shown as the mean ± SEM (*n* = 50). Asterisks are reported for cases with statistical significance: **** 24 h 1 G *Vs* 24 h μG, *p* = 0.0001. The statistical analysis of the data was performed using one-way analysis of variance (ANOVA) with Turkey’s test for multiple comparisons.

To deeper investigate the impact of simulated μG on VE-Cad protein mechanotransduction, in particular on the role mechanical unloading plays in the formation of VE-Cad-positive endothelial cell-cell junctions, immunofluorescence microscopy was performed. Specifically, the formation of a functional mature barrier, starting from a sub-confluent monolayer of HUVECs cultured in MOC, was followed and analysed over time. Cells were seeded in the MOC and allowed to grow overnight prior to 24 h of µG exposure (Time 0). At Time 0, VE-Cad pattern appeared intermitted with perpendicular dash-like structures forming between neighboring cells, indicating that premature formation of VE-Cad positive cell-cell junctions had formed post-seeding incubation (Fig. 6A). Cells exposed to 24 h of 1 G (control) conditions proliferated as expected and developed a continuous fluorescence pattern at the border of neighboring cells indicating a mature VE-Cad positive cell-cell junctions (Fig. 6B). Conversely, those cells exposed to 24 h of μG exhibited a similar VE-Cad localisation pattern to that of Time 0, suggesting that VE-Cad positive cell-cell junctions had not matured in a mechanically unloaded environment (Fig. 6C), as expected from previous result highlighting the suppressed production of the protein under μG condition (Fig. 5F). VE-Cad positive cell-cell junction formation was quantified for all conditions by plotting the fluorescence intensity profile of the cell boundary (Fig. 6D) and further calculating the percentage of VE-Cad positive intensity at the junctional border normalized by the mean cytoplasmic intensity. VE-Cad localization to cell-cell junctions in cells exposed to 24 h of µG significantly decreased by 74% when compared to cells exposed to control 1 G condition and 67% when compared to control Time0 (Fig. 6E). Taken together this data suggests that µG obstructs maturation of VE-Cad positive cell-cell junctions thus compromising the functionality of the vascular barrier.

### Prolonged exposure to microgravity induces actin-mediated endothelial cell junction opening

**Fig. 7:**
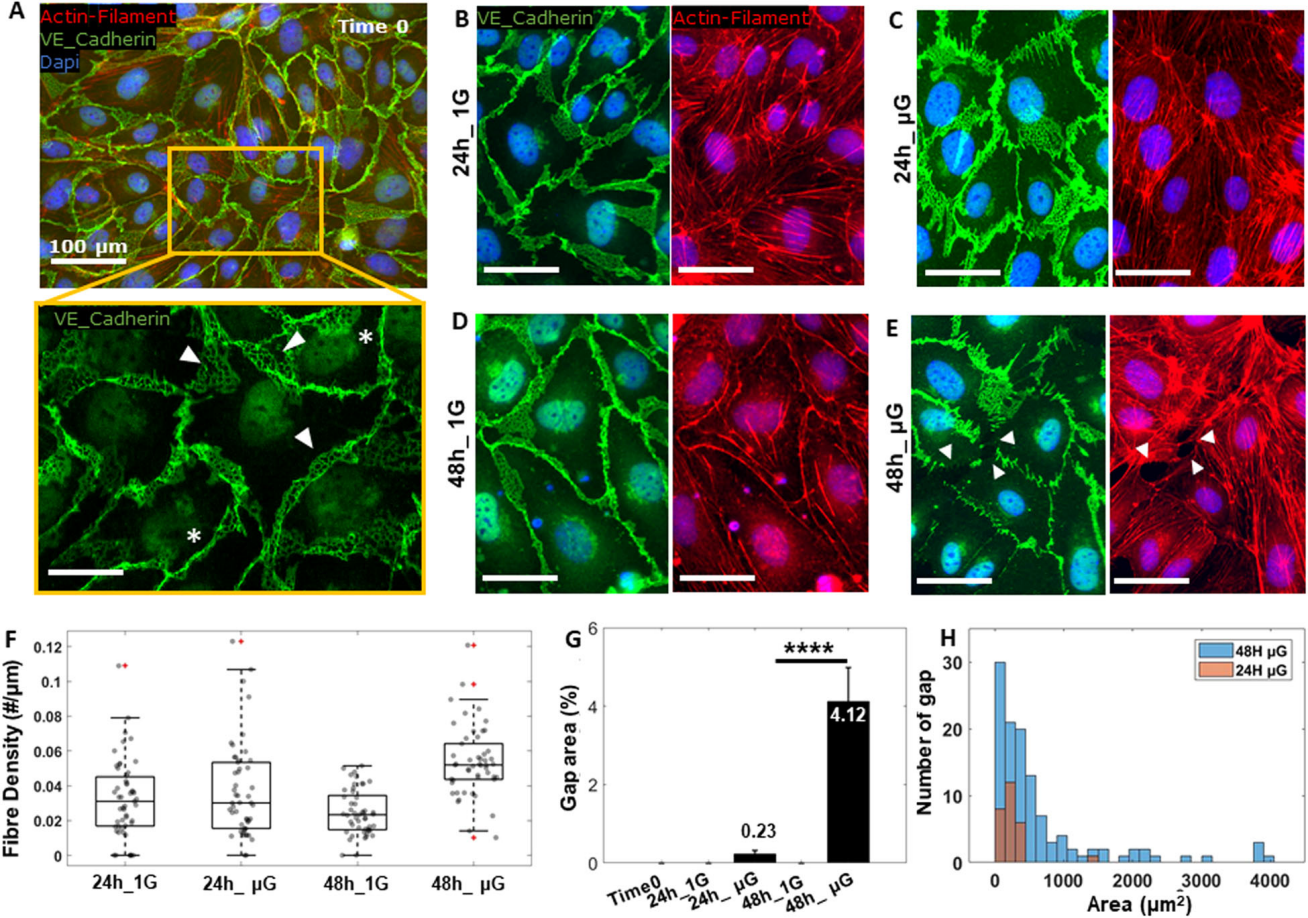
Junction opening and gap formation. Fluorescence images of a confluent monolayer of HUVECs showing Actin-Filament (red), VE-Cad (green) and Dapi (blue) at Time 0 (**A**) and after 24 h and 48 h of gravity (1 G) (**B**, **D**) and μG (**C**, **E**) conditions. Inset of Time 0 shows the heterogeneous patterns of VE-Cad protein clustered at cell border in reticular network (arrows) and linear configuration (stars). Intermitted VE-Cad pattern and gap formation in 48 h μG sample are also indicated with arrows. **F** Box plots and scatter data points for actin stress fibers density analyzed for all experimental conditions. **G** Histogram showing the percentage of opened gap area for all experimental conditions. **H** Histogram showing the distribution of gap in number and dimension for 24 h and 48 h microgravity group. Scale bars, 50 µm. Asterisks are reported for cases with statistical significance: **** 24 h μG *Vs* 48 h μG, *p* = 0.00004. The statistical analysis of the data was performed using one-way analysis of variance (ANOVA) with Turkey’s test for multiple comparisons.

We next sought to determine the influence of µG on mature endothelial cell-cell junctions. HUVECs were then grown to confluency and allowed to establish mature cell-cell junctions comprising of a phenotypic VE-Cad appearance with both reticular network and linear configuration^39^ (Fig. 7A, inset; arrows and asterisks respectively) prior to µG exposure. HUVECs with an established VE-Cad positive network showed no change in VE-Cad localization following 24 h of mechanical unloading when compared to 1 G control samples (Fig. 7B, C). However, those cells exposed to µG showed a different organization of VE-Cad positive signal with the junction assuming a perpendicular filamentous configuration, suggesting that exposure to µG may induce junctional remodeling at cell-cell borders. To further explore this hypothesis, we subjected confluent HUVEC monolayers to 48 h of mechanical unloading to determine if a longer exposure time would trigger a loss of VE-Cad from the junctional architecture. Cells exposed to 48 h of µG showed weak VE-Cad staining when compared to 1 G control cells and the formation of numerous actin stress fibers that traversed the cell body, not present in control cells (Fig. 7D, E). Stress fibers from the actin cytoskeleton are well known to be a key mechanotrasducers component involved in the remodeling of adherent’s junction at cell-cell border by redistributing the internal cellular forces within the cytoplasm when activated by mechanical stimuli^40,41^.

With an already established image analysis^34^, we quantified the density of stress fibers within HUVEC cell body after 24 and 48 h of 1 G and µG condition by using line scan tool in ImageJ software as explained in Method section. Interesting, as shown in Fig. 7F, the density of stress fibers within the cell bodies was found to be not significantly different in HUVECs exposed to 24 h of µG when compared to 1 G control, with a similar range of scattered data. On the other hand, HUVECs subjected to 48 h of µG condition expressed higher density of stress fibers compared to 1 G control sample, suggesting that µG may impact on junction stabilization by activating and gathering stress fibers within the cell body thus inducing a change in the functional state of the cell. Moreover, cells subjected to 48 h of µG displayed VE-Cad positive junctions as perpendicular, dash-like structures that crossed cell boundaries at sporadic intervals creating a discontinuous barrier (as indicated by arrows in Fig. 7E), while those cells exposed to 1 G control environments displayed the characteristic continuous reticular network and linear configuration (Fig. 7D). We further quantified the discontinuous VE-Cad positive contacts at cell-cell border by measuring the negative area in between contact points, termed here as ‘gap’, and found that the total gap area had significantly increased by 95% when compared to HUVEC cells exposed to 24 h of μG (Fig. 7G). When gaps were plotted as distribution in number and dimension (Fig. 7H) results also indicated that not only longer exposure of simulated μG may favor the formation of more gaps between cells but that gaps also increase in dimension. Overall, these results confirmed the implication that mechanical unloading has on endothelial cell mechanotransduction processes, suggesting that prolonged exposure to µG may trigger the molecular disassembly of VE-Cad from junctional structures via actin filaments rearrangement, ultimately resulting in a loss of biological barrier integrity and functionality.

## Discussion

The significance of advancing space mechanobiology research using μG simulators is due to the rare opportunity of conducting space mission on the International Space Station (ISS), yet the urgent need in understanding the role of mechanical forces and underlying mechanotransduction mechanisms driving severe terrestrial and space-related pathologies. Gravitational force represents a persistent inevitable constrain that drives cellular and molecular pathways to adopt specific configuration with a privilege direction^42^. Thus, by removing the gravitational field, living cells will have more degrees of freedom which can lead to the development of new cellular phenotypes and biochemical properties^43^. In other words, by exposing cells to a simulated mechanical unloading environment, researchers are able to unveil molecular mechanisms that would go otherwise undetected in normal gravity condition. This promising approach introduces the field of space mechanobiology in translational medicine with the perspective of revolutionizing our paradigm and knowledge in fundamental cell biology toward the development of medical countermeasure.

Currently, there are a limited number of microgravity simulators available, including 2-dimensional and 3-dimensional clinostats, rotating vessels and random positioning machines (RPM)^44^. The most common μG simulator, used for cell culture studies, is the RPM which works by continually providing random changes in X-Y plane orientation thus achieving the averaging of the gravity vector to zero over time^9^. While this approach has been widely used, it also introduces practical challenges that risk hampering the progress of the research, such as the usage of conventional culture plastic flasks that require large volume of media to avoid bubble formation and consequent shear stress experienced by the cells as well as the introduction of gas exchange gradient and possible contamination due to leakage. In this study, a fast and cheaper approach to develop biocompatible, gas permeable and reusable biological platforms, here named as Microgravity-on-a-chip (MOC), was developed and characterized to provide an alternative solution for conducting more reliable simulated μG experiments. Compared with conventional lab-on-chip fabrication method, which requires specific and expensive equipment to develop microfluidic master molds in SU-8 photoresist, the proposed MOC platform utilizes 3D printing procedures and uses sacrificial materials that allow users to customize the designs based on their experimental needs while saving in cost and time. The MOC has been conceived to have specific characteristics to combat the limitations in using conventional culture plastic flasks and plates coupled with the RPM simulator. A key advantage of the proposed MOC is the enclosed and sealable cell culture environment that only requires a cell medium volume in the order of microliters. Secondly, the use of syringes, tubing, and clamps allows for straightforward cell seeding protocol that avoids air bubble formation and therefore negates the possibility of unwanted shear stresses during RPM rotation. Moreover, the silicon structure provides a gas exchange surface all over the sample thus a homogeneous diffusion of optimal amount of oxygen throughout the chamber. Finally, the compactness allows the platform to be suitable for the critical geometry and sample position required in RPM experiments.

Our interest was then to leverage the simulated μG environment through the proposed MOC platform to investigate the most aggressive brain cancer, i.e., glioblastoma, searching for mechanosensitive molecules in both A-172 GBM cells and HUVEC cells that are involved in phenotypic and functional changes under such condition. We first realized MOCs with different volume to optimize the best culture condition. We then demonstrated MOCs biocompatibility and applicability with RPM rotating experiments by exposing both cell types to 24 h of simulated μG followed by several biological assays, including proliferation, viability, morphology, protein expression, and imaging of molecular structures. Our results indicated that both cell types are susceptible when the gravitational vector is disrupted, confirming the impact that μG has on both cancer and healthy cells functionality. For instance, A-172 GBM cells expressed a high cell viability after simulated μG condition, together with early-stage morphological changes and a suppressed proliferation activity, demonstrating ability to sense the lack of gravitational force and adapt to the new environment. These results are supported by previous studies showing the impact that μG has on cancer cell survival and functionality, such as cell adhesion, proliferation and migration^11–13,45–48^. To explore molecular targets able to respond to the mechanical unloading condition, we considered two mechanosensitive molecules involved in adhesion processes, namely YAP-1 and Vinculin proteins. When activated through the Hippo signaling pathway by external mechanical stimuli and structural features of the Extra Cellular Matrix (ECM), YAP-1 molecule acts as a transcriptional regulator for genes involved in proliferation mechanisms^47–49^, promoting the formation of Focal Adhesions (FAs)^35^, which are the mechanical links between actin filaments and the ECM^49^. Interesting, it has been previously shown that activation of YAP-1 in cancer cells, contributes to many oncogenic-associated mechanotransduction signaling pathways, including tumor initiation, progression, and metastasis^50^. Another molecule that is critically involved in cell migration and adhesion processes is Vinculin, which is a cytoskeletal protein associated with both cell-cell and cell-ECM junctions, where it anchors actin filaments and transmits mechanical signals to the ECM via FA^36,51,52^. As such, YAP-1 and Vinculin represent a molecular entry point to better understand the high sensitivity of cancer cells to the changes in mechanical properties of the external environment, including mechanical unloaded condition. Recent studies in different cancer cells have shown that simulated μG is able to alter the balance of forces at FA sites, where vinculin is enriched, inducing reduced proliferation ability and metastasis activity^11,12,53^. In line with these results, we found a reduction in both proteins expression when GBM cells were subjected to simulated μG for 24 h, suggesting that the mechanical unloaded condition compromised GBM proliferation mechanism due to the inactivation of the mechanosensitive YAP-1 protein followed by a lack of anchoring points to the actin cytoskeleton thus a change in cell structural stability. It is important to note that these results are consistent with our previous study^33^, where the evaluation of μG impact on the same GBM cells was conducted in conventional 96 well-plate, reinforcing the suitability and reproducibility of our biological platform for μG research.

Current treatment delivery strategies for GBM patients usually include the intravenous delivery of chemotherapeutic and as such they heavily rely on chemotherapeutics to cross the tightly regulated brain vasculature and be taken up by the hyperproliferative tumor cells. As a consequence of the highly selective biological barrier, current GBM treatment strategies remain inefficient and result in the high number of patients who experience cancer recurrence and death^25^. To model the vasculature in vitro, we cultured HUVECs in the MOC device and found them to be also highly sensitive to the simulated μG environments as proliferation was impaired and cell morphology altered, in line with previously published results^29–32,54^. For instance, Janmaleki et al.^30^ showed that HUVECs subjected to 24 h of μG exhibited a significant decrease in cell stiffness and viscosity as a result of cytoskeletal reorganization. Here, we reported that HUVECs exposed to 24 h of μG became rounded and exhibited a tortuous border, suggesting that actin cytoskeletal reorganization may occur as a first response to the adaptation to μG. However, the actin cytoskeleton plays an important role not only in determining cell shape and stiffness, but also in dictating the function and structure of endothelial barrier properties, such as cell-cell junctions^40^. The vascular endothelial cadherin (VE-Cad) junction is localized at the cell-cell border where dynamically interacts with other molecular complexes, including actin filaments, by transducing extracellular mechanical stimuli within the cytoplasm thus regulating vascular integrity and permeability^55,56^. In an intact mature endothelium monolayer, VE-Cad remodels into an overall linear and reticular pattern sustained by actin bundles along the perimeter of the cells that promote cell-cell contact and strengthen the barrier^39,57^. Under cell proliferation and migration processes, as well as inflammatory conditions, actin filaments remodel and rearrange in the form of stress fibers within the cytoplasm, indicating an activated cellular state^41^.

In an attempt to elucidate the role of μG in actin-mediated stabilization and maturation of junctions at cell-cell border we first subjected a growing sub-confluent monolayer to 24 h of mechanical unloaded condition. During junctional development at cells border, μG appeared to impede VE-Cad protein assembly at the junction complexes as expression levels of the 24 h period did not change when compared to Time 0 sample with a junction pattern resulting immature between endothelial cells. Moreover, although not significantly, we observed a slight decrease in vinculin protein expression, which is strictly correlated with mechanotransduction at cell-ECM site. Regarding a mature HUVEC monolayer with formed and intact junctions, we observed an initial remodeling of VE-Cad protein at cell-cell border after 24 h of μG exposure, followed by a significant junction disassembly and stress fibers reorganization with consequent gap formation under longer μG exposure, i.e., 48 h. Moreover, prolonged exposure of simulated μG favored the formation of bigger gaps between cells suggesting a reduced integrity and functionality of the biological barrier.

While a plethora of experimental evidence conducted on numerous cell types has demonstrated that under mechanical unloading cells present cytoskeletal reorganization^12,48,54,58–61^ followed by transcription and translation of cytoskeletal proteins^62^, very few studies focused on the mechanical unloading impact on VE-Cad molecule in endothelial cells and its role in barrier functionality^63,64^.

A significant contribution was provided by Shi et al.^64^, who found that fluorescence dye passage through a HUVEC monolayer cultured in trans well chamber was largely increased after 24 h of 2D clinostat-simulated μG condition. The study also proposed the possible molecular pathways responsible for the enhanced HUVECs permeability being the up-regulation of Ras-related protein 1 (RAP1) and the decrease activation of Ras-related protein 2 (RAP2), which are molecules that antagonistically regulate the adherents junction in endothelial cells^65^. Here, we further contributed to elucidate the implication of mechanical unloading on endothelial barrier functionality showing the impact that μG has on mature junctions with the formation of gap between cells. Further studies will explore this line of investigation to identify and characterize the actual molecular mechanisms responsible for vascular barrier “opening” for the development of strategies for effective targeted chemotherapeutics delivery. However, some limitations are also presented. For instance, in the contest of glioblastoma, brain vasculatures are formed of specialized brain endothelial cells, whose barrier functionality is also regulated by tight junctions (Z0-1) and other brain cells, including astrocytes and pericytes, which are not here investigated. Also, cell culture protocol has been performed in static condition while physiological flow is required to better mimic the in vivo condition of endothelial cells.

This study introduces and highlights the application of the MOC in advancing cell biology research under simulated μG condition, as a simple and easy-to-use alternative strategy. The features of the MOC and the ability to conduct basic and advanced molecular biology assays were demonstrated by unveiling insights into the underlying mechanotransduction response of glioma and endothelial cells to the mechanical unloaded condition. This demonstrates the value and impact of space mechanobiology in understanding healthy and cancerous cell survival and function setting the foundation for a good comparison. With further advancement in cell mechanobiology research, μG will undoubtedly play a critical role as an invaluable tool for future discoveries. As such, continuous developments into complementing technologies are required for support μG research. Future improvements of the MOC platform will seek to introduce different 3D cell culture into the platform to mimic different microenvironments and stiffness as well as the introduction of fluidic pump systems that can replicate more closely the cell physiological conditions and support long-term culture into the MOC during RPM operation. In summary, we encourage the application of the described MOC platform as a valuable and reliable tool for cellular μG research and an excellent starting point for the acceleration of discoveries in the field of molecular therapeutic approaches for disease treatment.

## Supplementary information

Reporting Summary

## Data Availability

The data supporting the conclusion of this article will be made available by the Corresponding author, without undue reservation.
