# Supplementary material for: Testing 3D printed biological platform for advancing simulated microgravity and space mechanobiology research
Source: NPJ Microgravity. 2022 Jun 3;8:19. doi: 10.1038/s41526-022-00207-6 (PMC9166742; doi:10.1038/s41526-022-00207-6)
Supplement: Supplementary file 1 — Reporting Summary [file 41526_2022_207_MOESM1_ESM.pdf]

nature portfolio

Corresponding author(s): Giulia Silvani

Last updated by author(s): 18/03/2022

# Reporting Summary

Nature Portfolio wishes to improve the reproducibility of the work that we publish. This form provides structure for consistency and transparency in reporting. For further information on Nature Portfolio policies, see our Editorial Policies and the Editorial Policy Checklist.

## Statistics

For all statistical analyses, confirm that the following items are present in the figure legend, table legend, main text, or Methods section.

| n/a | Confirmed | |
|---|---|---|
| ☐ | ☒ | The exact sample size ($n$) for each experimental group/condition, given as a discrete number and unit of measurement |
| ☐ | ☒ | A statement on whether measurements were taken from distinct samples or whether the same sample was measured repeatedly |
| ☐ | ☒ | The statistical test(s) used AND whether they are one- or two-sided<br>*Only common tests should be described solely by name; describe more complex techniques in the Methods section.* |
| ☒ | ☐ | A description of all covariates tested |
| ☒ | ☐ | A description of any assumptions or corrections, such as tests of normality and adjustment for multiple comparisons |
| ☒ | ☐ | A full description of the statistical parameters including central tendency (e.g. means) or other basic estimates (e.g. regression coefficient) AND variation (e.g. standard deviation) or associated estimates of uncertainty (e.g. confidence intervals) |
| ☒ | ☐ | For null hypothesis testing, the test statistic (e.g. $F$, $t$, $r$) with confidence intervals, effect sizes, degrees of freedom and $P$ value noted<br>*Give P values as exact values whenever suitable.* |
| ☒ | ☐ | For Bayesian analysis, information on the choice of priors and Markov chain Monte Carlo settings |
| ☒ | ☐ | For hierarchical and complex designs, identification of the appropriate level for tests and full reporting of outcomes |
| ☒ | ☐ | Estimates of effect sizes (e.g. Cohen's $d$, Pearson's $r$), indicating how they were calculated |

*Our web collection on statistics for biologists contains articles on many of the points above.*

## Software and code

Policy information about availability of computer code

| | |
|---|---|
| Data collection | N/A |
| Data analysis | N/A |

For manuscripts utilizing custom algorithms or software that are central to the research but not yet described in published literature, software must be made available to editors and reviewers. We strongly encourage code deposition in a community repository (e.g. GitHub). See the Nature Portfolio guidelines for submitting code & software for further information.

## Data

Policy information about availability of data

All manuscripts must include a data availability statement. This statement should provide the following information, where applicable:

- Accession codes, unique identifiers, or web links for publicly available datasets
- A description of any restrictions on data availability
- For clinical datasets or third party data, please ensure that the statement adheres to our policy

The data supporting the conclusion of this article will be made available by the author, without undue reservation

# Field-specific reporting

Please select the one below that is the best fit for your research. If you are not sure, read the appropriate sections before making your selection.

☒ Life sciences ☐ Behavioural & social sciences ☐ Ecological, evolutionary & environmental sciences

For a reference copy of the document with all sections, see nature.com/documents/nr-reporting-summary-flat.pdf

# Life sciences study design

All studies must disclose on these points even when the disclosure is negative.

| | |
|---|---|
| Sample size | Average values of at least three independent experiments ± SEM are showed for viability, proliferation and protein quantification assays. Morphological parameters, junction evaluation and actin density were measured from 50 cells in 8 different fluorescence images for each experimental condition while gaps quantification has been performed on 30 different fluorescence images for each experimental condition. |
| Data exclusions | N/A |
| Replication | The statistical analysis of the data was performed using one-way analysis of variance (ANOVA) with Turkey's test for multiple comparisons, using GraphPad Prism (v 7.04). P-value is reported for statistical significance. Comparisons between samples were considered to be statistically significant if the p-value was *p < 0.05, **p < 0.01, ***p < 0.001, **** p<0.0001. |
| Randomization | N/A |
| Blinding | N/A |

# Reporting for specific materials, systems and methods

We require information from authors about some types of materials, experimental systems and methods used in many studies. Here, indicate whether each material, system or method listed is relevant to your study. If you are not sure if a list item applies to your research, read the appropriate section before selecting a response.

## Materials & experimental systems

| n/a | Involved in the study | |
|---|---|---|
| ☐ | ☒ | Antibodies |
| ☒ | ☐ | Eukaryotic cell lines |
| ☒ | ☐ | Palaeontology and archaeology |
| ☒ | ☐ | Animals and other organisms |
| ☒ | ☐ | Human research participants |
| ☒ | ☐ | Clinical data |
| ☒ | ☐ | Dual use research of concern |

## Methods

| n/a | Involved in the study | |
|---|---|---|
| ☒ | ☐ | ChIP-seq |
| ☒ | ☐ | Flow cytometry |
| ☒ | ☐ | MRI-based neuroimaging |

## Antibodies

| | |
|---|---|
| Antibodies used | Primary antibodies against VE-Cadherin (Abcam, Cat. No. ab33168), active (non-phosphorylated) Yap-1 (Abcam, Cat. No. ab205270) and Vinculin (Sigma-Aldrich, Cat. No. V9131) were used for IWB and IF assays |
| Validation | Human VE Cadherin peptide (ab27462): Used for WB and IF at concentration of 1 µg/ml. Abcam recommends using BSA blocking with this product.<br>Human active (non-phosphorylated) Yap-1 (ab205270): Used for WB at concentration of 1:500. Abcam recommends using BSA blocking with this product.<br>Anti-Vinculin antibody, Mouse monoclonal (V9131): Used for IWB at concentration of 1:200 using extract of human fibroblasts |
